# The psychological determinants of internet gaming disorder: Vulnerability to stress, psychological well-being, and comorbidity

**DOI:** 10.1192/j.eurpsy.2021.458

**Published:** 2021-08-13

**Authors:** J. Cardoso, T. Ferreira, A.R. Dores

**Affiliations:** 1 Department Of Social And Behavioural Sciences, University Institute of Maia, Maia, Portugal; 2 Laboratory Of Neuropsychophysiology (fpceup); Center For Rehabilitation Research (ess-p.porto), Faculty of Psychology and Education Sciences; School of Health, Polytechnic of Porto, Porto, Portugal

**Keywords:** PSYCHOLOGICAL WELL-BEING, comorbidity, Internet Gaming Disorder, Vulnerability to Stress

## Abstract

**Introduction:**

A variety of psychological determinants, such as vulnerability to stress, low levels of psychological well-being and several comorbidities, have been hypothesized to play a role in the development, and maintenance of Internet Gaming Disorder (IGD). However, evidence has been insufficient to sustain an overarching model of the causal pathways leading to IGD.

**Objectives:**

. This study aimed to depict a model of the causal links between vulnerability to stress, psychological well-being, and symptoms of common mental disorders (e.g., depression, generalized anxiety, phobic anxiety, obsessive-compulsive disorder, somatization, and hostility).

**Methods:**

. A community-based sample of Portuguese gamers (N = 153; Mage = 21.92; 15.29% female) completed measures of IGD (IGDS9-SF), mental health (SCL-90-R), psychological well-being (EBEP), and vulnerability to stress (23QVS). A machine learning algorithm – Greedy Fast Causal Inference – was used to infer a model of the causal pathways linking those psychological determinants to IGD.

**Results:**

. Hostility and psychological well-being were directly involved with a subgroup of IGD symptoms (i.e., gaming used as escape, tolerance, withdrawal, and loss of control). Stress vulnerability and symptoms of mental disorders were only indirectly implicated in the causal pathways leading to IGD.

**Conclusions:**

. It is likely that several psychological factors implicated in the causal pathways leading to IGD, have not been yet identified. Future research should directly test specific models of the causal pathways involved in the development and maintenance of IGD symptoms.

**Disclosure:**

No significant relationships.

